# The Efficacy of Zinc Phthalocyanine Nanoconjugate on Melanoma Cells Grown as Three-Dimensional Multicellular Tumour Spheroids

**DOI:** 10.3390/pharmaceutics15092264

**Published:** 2023-08-31

**Authors:** Nkune Williams Nkune, Heidi Abrahamse

**Affiliations:** Laser Research Centre, Faculty of Health Sciences, University of Johannesburg, Doornfontein, P.O. Box 17011, Johannesburg 2028, South Africa; nkune.williams@gmail.com

**Keywords:** photodynamic therapy, zinc phthalocyanine, gold nanoparticles, three-dimensional multicellular tumour spheroids

## Abstract

Melanoma remains a major public health concern that is highly resistant to standard therapeutic approaches. Photodynamic therapy (PDT) is an underutilised cancer therapy with an increased potency and negligible side effects, and it is non-invasive compared to traditional treatment modalities. Three-dimensional multicellular tumour spheroids (MCTS) closely resemble in vivo avascular tumour features, allowing for the more efficient and precise screening of novel anticancer agents with various treatment combinations. In this study, we utilised A375 human melanoma spheroids to screen the phototoxic effect of zinc phthalocyanine tetrasulfonate (ZnPcS_4_) conjugated to gold nanoparticles (AuNP). The nanoconjugate was synthesised and characterised using ultraviolet-visible spectroscopy, a high-resolution transmission electron microscope (TEM), dynamic light scattering (DLS), and zeta potential (ZP). The phototoxicity of the nanoconjugate was tested on the A375 MCTS using PDT at a fluency of 10 J/cm^2^. After 24 h, the cellular responses were evaluated via microscopy, an MTT viability assay, an ATP luminescence assay, and cell death induction using annexin propidium iodide. The MTT viability assay demonstrated that the photoactivated ZnPcS_4_, at a concentration of 12.73 µM, caused an approximately 50% reduction in the cell viability of the spheroids. When conjugated to AuNPs, the latter significantly increased the cellular uptake and cytotoxicity in the melanoma spheroids via the induction of apoptosis. This novel Zinc Phthalocyanine Nanoconjugate shows promise as a more effective PDT treatment modality.

## 1. Introduction

Cancer is a dreadful disease caused by the formation and growth of atypical cells that proliferate erratically [1]. Melanoma is the most perilous form of skin cancer, arising from melanocytes, and it is notorious for its inevitable multidrug resistance, ease of relapse, and poor prognosis [2]. When identified early, it is possible to eradicate melanoma through surgical excision, leading to better survival rates. However, once metastasis occurs, traditional therapies, such as radiotherapy, chemotherapy, and immunotherapy, provide negligible therapeutic benefits to melanoma patients [3]. Moreover, these treatments have limitations such as a poor bioavailability and off-target toxicity, killing neighbouring healthy cells [1]. Melanoma resistance to therapies is also attributed to the intrinsic heterogeneity of tumours, which promotes their survival and aggressiveness [4]. In addition, there is a scarcity of specific genetic mutations that are targetable by currently available treatment modalities, limiting the alternatives for melanoma treatment [5]. Melanoma cells not only activate the resistance pathways that counteract the cell death induced by therapies, but also have the ability to evade immune system barriers [6]. Furthermore, one of the major challenges in melanoma treatment is poor drug penetration, which leads to the partial treatment of cancer cells due to the unique features of melanomas in relation to blood vessel structure and tissue architecture [7]. In recent years, researchers have been actively engaged in incorporating nanomaterials to target cancer cells and enhance the bioavailability of anticancer agents in targeted tissues [8].

Photodynamic therapy (PDT) is a relatively new cancer therapy, which incorporates a light source, photosensitizer (PS), and oxygen molecules. A PS molecule absorbs light at an appropriate wavelength, initiating the photochemical processes that ultimately generate cytotoxic reactive oxygen species (ROS), which trigger necrosis or apoptosis [9]. Light is essentially indispensable for PDT applications, and therefore it should effectively penetrate biological barriers without any deterrent and activate a PS absorbed by tumour cells. Biological tissues poorly absorb light between 650 and 800 nm, and therefore this region is referred to as the biological tissues’ optical window [10]. However, in the case of melanoma, the dynamics are different due to the presence of a high melanin content. Studies have highlighted that melanin promotes melanoma resistance to PDT by attenuating the amount of light dose aimed at tumour sites [5,6]. Melanin absorbs light throughout the entire therapeutic window region (500–600), which is crucial for PDT, making it a major antagonist of PS for light absorption. Furthermore, studies have reported that melanin serves as an antioxidant and ROS scavenger, promoting melanoma resistance to PDT [11].

Zinc phthalocyanine derivatives have gained significant attention in PDT due to their unique advantages, such as minimal toxicity, ease of synthesis, rapid and selective accumulation in targeted tissues, and strong absorption at a 670 nm wavelength, which allows for the treatment of deep-rooted tumours [12]. Zinc phthalocyanine tetrasulfonate (ZnPcS_4_), one of the phthalocyanine PSs, has been extensively investigated in PDT research due to its hydrophilic nature, attributed to the presence of sulphate groups [12]. Additionally, it displays high quantum ROS yields in the 680 nm range with an improved tissue penetration depth [13].

Despite the numerous attributes of PDT in cancer treatment, PDT still presents a series of drawbacks that limit its capacity to effectively obliterate cancer cells [14]. Most conventional PSs are inherently hydrophobic with a poor solubility in water. Therefore, they tend to aggregate when subjected to aqueous conditions, which considerably hampers their quantum yields of ROS generation [15]. Furthermore, studies have pointed out that conventional PSs are degraded by immune system checkpoints upon entering the body, drastically reducing their bioavailability in cancerous tissues [16]. Such concerns have led to the modification of PSs with nanomaterials to enhance their bioavailability, increase their tumour affinity, and allow for a deeper tumour penetration, thus increasing the treatment potency and reducing off-target toxicity [17].

Gold nanoparticles (AuNPs) are endowed with extraordinary physiological and optical properties ideal for PDT applications [18]. They have a high loading capacity, remarkable stability, high scattering energy, high biocompatibility, maximum absorption within the near infrared region, and facile surface functionalisation. In addition, AuNPs not only preserve PSs from enzymatic degradation, but also increase their cellular uptake load via the enhanced permeability and retention (EPR) effect, since they are small, hydrophilic structures that can evade immune system barriers [18]. Studies have reported that, when zinc phthalocyanines are functionalised with AuNPs, they produce longer triplet lifetimes than bare PSs, ultimately increasing the overall efficacy of PDT [19].

Virtually all PDT data are still obtained from traditional two-dimensional (2D) cell culture models, which do not sufficiently resemble the physiological conditions and three-dimensional (3D) architecture of native tumour cells [20]. As a result, a significant discrepancy has been noted when transferring results from 2D cell cultures into in vivo tumour models [21]. Additionally, misleading data from 2D cell cultures often lead to inaccurate predictions of drug efficacy and toxicity, which ultimately delay the processes for validating and approving new anticancer agents [20]. Thus, there is an urgent need for better in vitro cell culture models for screening PS drugs before using in vivo animal models.

Multicellular tumour spheroids (MCTS) are excellent 3D models that can bridge the gap between in vitro studies and animal models, since they replicate native tumours morphologically and biologically [22]. They exhibit the properties of solid tumours, such as cellular heterogeneity, the distribution of oxygen and nutrients, cellular signalling mechanisms, cellular interactions, growth kinetics, and therapeutic resistance patterns similar to those observed in patients [20]. Currently, preliminary investigations of nanocarrier-mediated delivery systems are typically conducted on 2D cell culture models. While convenient and simple to prepare, 2D cell cultures discover promising drug formulations that do not reflect in vivo responses in animal models or clinical studies [23]. As a result, several drawbacks related to NP formulations are normally identified during the later stages of drug development, delaying the process of discovering new drugs [24]. To some extent, these discrepancies in therapeutic outcomes could be attributed to the inability of 2D cell cultures to accurately simulate extracellular barriers [24]. While NPs administered to a monolayer cell culture typically accumulate in cells without any physical obstruction, the diffusion of NPs delivered in vivo would be counteracted by the ambiguous tumour-associated extracellular matrix (ECM) [11]. The 3D architecture of a tumour mass also significantly influences the diffusion characteristics of drugs, both via cell-to-cell and cell-to-matrix interactions [24]. Therefore, screening anticancer agents using 3D cell culture models holds great promise for improving the preclinical testing of drug candidates to improve upon new pharmacological approaches, as well as for providing valuable information for designing in vivo studies [25].

In this present study, gold nanoparticles, AuNP-PEGs, were synthesised and incorporated with a ZnPcS_4_ PS to enhance its cellular uptake and efficacy in A375 MCTS. Ultimately, the phototoxic effect of the ZnPcS_4_-AuNP was compared to that of bare ZnPcS_4_.

## 2. Materials and Methods

### 2.1. Materials

Gold (III) chloride trihydrate (HAuCl_4_·3H_2_O, ≥99.9% trace metals basis), tri-sodium citrate (for molecular biology, ≥99%), tannic acid (ACS reagent), SH-PEG2k-NH_2_, Triton X-100 (T9284), Accumax™ solution (A7089), Foetal Bovine Serum (FBS), Amphotericin-β, Penicillin-Streptomycin, and Dulbecco’s Modified Eagle’s Medium (D5796) were all procured from Sigma-Aldrich, Johannesburg, South Africa. TrypLE^TM^ Select Enzyme (1X) (12563-029) ThermoFisher, Johannesburg, South Africa). Caspase 3, 8, and 9 Multiplex Assay kit (Ab219915) and 96-well ultra-low attachment plates (174929) were also purchased from ThermoFisher, Johannesburg, South Africa. Annexin V/PI apoptosis detection kit (556570) was procured from BD Biosciences, The Scientific Group, Johannesburg, South Africa).

### 2.2. Preparation of ZnPcS_4_

To prepare a working solution with a concentration of 125 µM, 0.0006 g of ZnPcS_4_ powder (SantaCruz^®^ Biotechnology sc-264509A, Johannesburg, South Africa) was solubilised in 1.25 mL of 0.001 M of phosphate-buffered saline (PBS) (Sigma-Aldrich, Johannesburg, South Africa) to form a stock solution of 0.0005 M, which was further diluted by adding 4 mL of PBS. The prepared solution was covered with foil and stored at room temperature.

### 2.3. Synthesis of Citrate-Au NPs

A total of 1 mL of 1% AuHCl_4_·3H_2_O was added to 79 mL of Millipore water in a three-neck flask under a reflux system, and subsequently, a solution of 0.5 mL of tannic acid 1%, 4 mL of 1% tri-sodium citrate, and 15.5 mL of Millipore water was added. The mixture was stirred at 60 °C for a few minutes to form a red-coloured solution of citrate-capped AuNPs. The synthesised NPs were stored at 4 °C for further experiments. 

### 2.4. PEGylation of AuNPs

In order to covalently immobilise SH-PEG-NH_2_ onto the surface of the AuNPs, 20 mg/mL of the PEG in PBS was added to 1 mL of citrate-capped AuNPs to establish a ligand exchange reaction. The solution was stirred at room temperature for a few minutes and incubated for 2 h. Subsequently, the solution was spun down to eliminate excess SH-PEG-NH_2_, and the pellet was weighed to determine the concentration of the pegylated AuNPs. Afterwards, the AuNPs were resuspended in PBS. Furthermore, standard concentrations of AuNPs, ranging from 20 to 200 µg/mL, were prepared using UV-Vis spectrophotometry at 520 nm to measure their absorbance, and a standard curve was generated from the obtained data to estimate the concentration of AuNPs bound to ZnPcS_4_, using the y = 0.0006x − 0.0131 equation.

### 2.5. Conjugation of ZnPcS_4_ to PEGylated AuNPs

In total, 1 mL of AuNP-SH-PEG-NH_2_ with an estimated concentration of 1 mg/mL was mixed with 1 mL of 125 µM of ZnPcS_4_ in a microtube covered with foil to protect the solution from light exposure. The solution was agitated at room temperature for 24 h using a multifunction vortex mixer (DAIHAN-brand MVM-10, Celsius) agitating at a 1500 rpm speed. The next day, the solution was subjected to vigorous centrifugation at 15,200 rpm for one hour so that the AuNPs bound to the ZnPcS_4_ could be fruitfully pelleted out of the solution. Thereafter, the supernatant was discarded to remove any unconjugated ZnPcS_4_. The resultant pellet, comprising ZnPcS_4_ conjugated to AuNP-SH-PEG-NH_2_, was resuspended in 1 mL of 0.001 M PBS and subjected to elementary characterisation techniques. This nanoconjugate was kept at 4 °C when not in use.

### 2.6. Determination of ZnPcS_4_ Loading Efficiency in AuNPs

The concentration of the ZnPcS_4_ bound to the AuNPs (loading efficiency) was determined by measuring the absorbance of the nanoconjugate (ZnPcS_4_-AuNP) at 673 nm. A standard calibration curve was generated using the absorbance values of different concentrations of ZnPcS_4_, ranging from 10 to 60 µM, at 673 nm. A linear equation (y = 0.0013x + 0.0017, regression, 0.9887) obtained from the standard curve was used to calculate the amount of ZnPcS_4_, whereby y represents the known absorbance of the sample at 673 nm, allowing for the x concentration to be calculated. Furthermore, the loading efficiency was calculated using the below formula.

$$\mathrm{PLE}\;(\%)=\frac{weight\;of\;loaded\;PS}{weight\;of\;PS\;in\;feed}\;\times \;100$$

### 2.7. Characterisation of the Nanoconjugate

#### 2.7.1. Determination of Spectroscopic Properties Using UV-Vis Spectrophotometry 

The spectroscopic properties were analysed using a Jenway Genova Nano Plus Life Science Spectrophotometer (Cole-Parmer Ltd., Stone, Staffordshire, UK) by scanning the individual components and nanoconjugate from 300 to 800 nm at 1 nm wavelength intervals, in order to validate their absorption peaks at distinctive wavelengths. All the samples were measured against 0.001 M PBS in a 1 mL UV fused quartz cuvette. The spectrum results were recorded and then represented on a line graph for analysis.

#### 2.7.2. Confirmation of Functional Groups Using Fourier Transform Infrared Spectroscopy

Fourier transform infrared spectroscopy was carried out using the potassium bromide (KBr) pellet technique. Briefly, 100 µL of each sample was stored overnight in a −80 °C freezer to freeze. The next day, the frozen samples were subjected to a freeze dryer for 3 days to solidify. Subsequently, the solidified samples were crushed into a powder, which was then mixed with KBr and compressed using a hydraulic press to generate a pellet. This pellet was then loaded onto the FTIR sample holder. The FTIR (Perkin Elmer Spectrum 100 FTIR spectrometer, University of Johannesburg, Analytical Chemistry Department) recorded the results at frequencies ranging from 400 to 4000 cm^−1^, with 25 scans.

#### 2.7.3. Dynamic Light Scattering (DLS) and Zeta Potential

The synthesised nanoconjugate and its individual components were characterised for their size and surface charge using the Malvern Zetasizer Nano ZS (Malvern Instruments, Zetasizer software, 7.03, Malvern, UK), which has a 4 mW He-Ne laser with a 633 nm wavelength. The samples were diluted in deionised water and sonicated to form a homogenous solution. The solution was then pipetted into a scratch-free plastic Zeta 3 × 3 mm dip cell cuvette with a built-in electrode for DLS and Zeta measurements. The diluted samples were finally run at 25 °C, using 13° and 173° angles.

#### 2.7.4. Investigation of Particle Size and HRTEM with Energy-Dispersive X-ray Spectroscopy (EDS) Analysis

A JEM-2100 High Resolution Transmission Electron Microscope (HR-TEM) (JEOL Ltd., Tokyo, Japan) was used to determine the morphology and size of the nanoconjugates. The samples were sonicated for 15 min and loaded, in a dropwise manner, onto carbon-coated 200-mesh Cu TEM grids (Lot# 1261229, SPI Supplies). The grids were left to dry overnight in the dark. Thereafter, the dried grids were loaded onto the microscope, and the images were captured and measured for their size using ImageJ v1.53 software (National Institutes of Health and Laboratory for Optical and Computational Instrumentation (LOCI), University of Wisconsin, Madison, WI, USA). The same instrument is endowed with an energy-dispersive X-ray spectroscopy (EDS) device for detecting individual elements present on the Cu grids. The EDS spectroscopic method allowed for both elemental and qualitative studies of the compounds found in the sample. Hence, this feature was incorporated to validate the specific element forming the compounds.

### 2.8. Multicellular Tumour Spheroid Culture (MCTS)

Human melanoma cells (A375) purchased from cellonex were cultivated in DMEM media enriched with 10% Fetal Bovine Serum (FBS) and 0.1% of both penicillin-streptomycin and amphotericin-β. The A375 cells were cultured in a T-75 flask and incubated at 37 °C, with 5% CO_2_. Upon reaching 80% fluence, the cells were harvested from the T-75 tissue flask and seeded in an ultra-low attachment 96-well plate at a density of 4000 cells per well in 200 μL. They were incubated for 72–96 h until the MCTS reached an average diameter of 450 μm. 

### 2.9. Cellular Uptake of ZnPcS_4_-AuNP

The A375 MCTS were incubated with 12.73 µM of free ZnPcS_4_ and 12.73 µM of ZnPcS_4_-AuNP for 24 h at 37 °C in the dark. The spheroids were washed twice with HBSS and dissociated using a 200 µL detergent solution (0.1 M NaOH + 0.1% SDS) followed by repeated pipetting. Thereafter, the solubilised cells were centrifuged at 10,000 rpm for 10 min, and the supernatant was collected to measure the absorbance of the ZnPcS_4_ at 673 nm using a UV-vis spectrophotometer, which determined the concentration of the ZnPcS_4_ by the means of a standard curve. The protein content of each sample was determined using the BCA protein kit, and the cellular uptake was normalised to the protein content.

### 2.10. Drug Release of ZnPcS_4_-AuNP

A total of 100 µL of ZnPcS_4_-AuNP was divided into aliquots and diluted with 900 µL of PBS buffer at two different pHs (7.4 and 5.4) to embody the physiological pH of a tumour. The contents were incubated at 37 °C for varying periods of time (0, 1, 4, 8, 12, 24, and 30 h), after which, they were subjected to centrifugation at 18,000 rpm for 10 min. The supernatants were collected, and the absorbance of the ZnPcS_4_ was measured. The concentration of the released ZnPcS_4_ was plotted with respect to the corresponding time, and the free ZnPcS_4_ was used as a reference.

### 2.11. Localisation of Nanoconjugate in MCTS

The A375 MCTS were incubated with predetermined concentrations of 12.73 μM of free ZnPcS_4_ and 12.73 μM of ZnPcS_4_-AuNP for 24 h at 37 °C. Thereafter, the spheroids were washed three times with HBSS. Carl Zeiss AXio Z1 fluorescence microscopy (Oberkochen, Germany) was used to capture the spheroid fluorescent sections up to a 360 μm distance from the periphery using the Alexa 594 filter. The fluorescence intensity of the captured images was quantified using Image J software.

## 3. IC_50_ Concentration Determination and ZnPcS_4_ Nanoconjugate-Mediated PDT Assays

In order to determine an optimal concentration of ZnPcS_4_ that could decrease cell viability by approximately 50%, the spheroids were incubated for 24 h with increasing concentrations of ZnPcS_4_, ranging from 1 to 20 µM, with and without laser irradiation. The spheroids to be irradiated were subjected to laser irradiation at a 673 nm diode laser with a fluence of 10 J/cm^2^ and an output power of 80 mW (light intensity: approximately 9 mW/J/cm^2^). Thereafter, post-PDT incubation, the IC_50_ concentration was calculated using a sigmoidal graph, and the obtained concentration was used throughout the study in both the free ZnPcS_4_ and ZnPcS_4_-AuNP treatments to validate the enhancement of PSs with nanoparticles.

### 3.1. Cell Viability

To determine the spheroid viability, the MCTS from the control and experimental groups were washed twice with PBS. The MCTS were transferred into microcentrifuge tubes. Firstly, the MCTS were dissociated with 200 μL of Accumax™ solution at 37 °C with continuous stirring for 20 min, followed by repeated pipetting. The MCTS were then centrifuged at 2500 rpm for 5 min to form a pellet. The cells were then resuspended in serum-free media. Thereafter, 100 μL of each cell suspension was transferred to a clear 96-well plate, and 0.5 mg/mL of MTT substrate was added to each well and incubated for 4 h. After 4 h of incubation, 100 μL of a solubilisation buffer was added and incubated for 24 h. The absorbance of the resulting solution was read at 540 nm with the VICTOR Nivo^®^ multi-mode plate reader (PerkinElmer, HH35940080 EN, Madrand, South Africa). The MTT results were used to determine the IC50 concentration of the ZnPcS_4_ using a sigmoidal fitting/dose response curve (origins). 

### 3.2. Adenosine Triphosphate (ATP)

The CellTiter-Glo^TM^ 3D luminescence (Promega, G968A, Madison, WI, USA) Kit was used to determine the intracellular ATP content of the MCTS. Briefly, the MCTS were transferred into microcentrifuge tubes. The MCTS were disintegrated via incubation with 200 μL of Accumax solution at 37 °C for 20 min, followed by continuous agitation. Thereafter, 200 μL of HBSS was added to attenuate the reaction. The tubes were then subjected to centrifugation at 2500 rpm for 5 min, and the supernatant was discarded, after which, the cells were resuspended in HBSS. In total, 100 μL of each cell suspension was transferred to an opaque 96-well plate, an equal volume of the ATP substrate was added, and the contents were agitated for 5 min to facilitate reagent penetration, lysis, and ATP recovery. The samples were incubated at room temperature for an additional 25 min, and the intracellular ATP luminescence was recorded using Victor Nivo^®^ multimode plate reader (Perkin-Elmer, Midrand, South Africa).

### 3.3. Morphology

A Wirsan Olympus CKX 41 invited light microscope was used to observe the morphological characteristics of the MCTS and their structural changes 24 h after PS- or nanoconjugate-mediated PDT, and images were captured using a digital camera Wirsam, Olympus CKX41, Johannesburg, South Africa.

### 3.4. Live/Dead Assay

The control and experimental MCTS were washed three times with PBS and stained with 1 μg/mL of ethidium bromide (EtBr) in conjunction with the same concentration of acridine orange (AO) for 5 min in PBS. Thereafter, the MCTS were rinsed three times with PBS and visualised using Alexa fluor 488 and EtBr channels under a Carl Zeiss fluorescent microscope using the Zen Pro (3.7) Carl Zeiss software.

### 3.5. Cell Death

To determine the mode of cell death induction, 24 h after treatment with the PS or nanoconjugate in the dark or after laser irradiation, the MCTS were dissociated with Accumax. Single-cell suspensions from the control and experiment groups were centrifuged, and the supernatants were discarded. The cells were then washed twice with ice-cold PBS and resuspended in an ice-cold 1× binding buffer. In total, 100 μL of the cell suspension was transferred to a flow cytometry tube and incubated with 5 µL of Annexin V-FITC and 5 µL of propidium iodide stains in the dark. The contents were gently vortexed and incubated at room temperature for 15 min in the dark. The cell preparations were analysed using a Becton Dickinson (BD) Accuri C6 flow cytometer (Franklin Lakes, NJ, USA) after adding 400 µL of 1× binding buffer to each tube.

### 3.6. Fluorometric Quantification of Caspase-3, 8, and 9 Activities 

Twenty-four hours after the PDT treatment, the single-cell suspensions of the various control and experimental groups were mixed with caspase solution in a poly-D-lysine-coated plate, followed by 1 h of incubation at room temperature in the dark. The PerkinElmer VICTOR Nivo^TM^ was used to measure the fluorescence at specific wavelengths: Ex/Em = 535/620 nm (Caspase 3), Ex/Em = 490/525 nm (Caspase 8), and Ex/Em = 370/450 nm (Caspase 9). The results for each experimental group were reported as a fold increase in caspase levels versus the untreated control.

### 3.7. Statistical Analysis

The differences between the control and experimental groups were tested using a one-way analysis of variance (ANOVA) with Dunnett test using Sigma Plot version 12. All the results are presented as mean ± standard error obtained from three independent experiments and *p* < 0.05 was considered to be statistically significant (*p* < 0.05 *, *p* < 0.01 **, and *p* < 0.001 ***).

## 4. Results

### 4.1. Characterisation

#### 4.1.1. UV/Visible Spectral Analysis 

**Figure 1:**
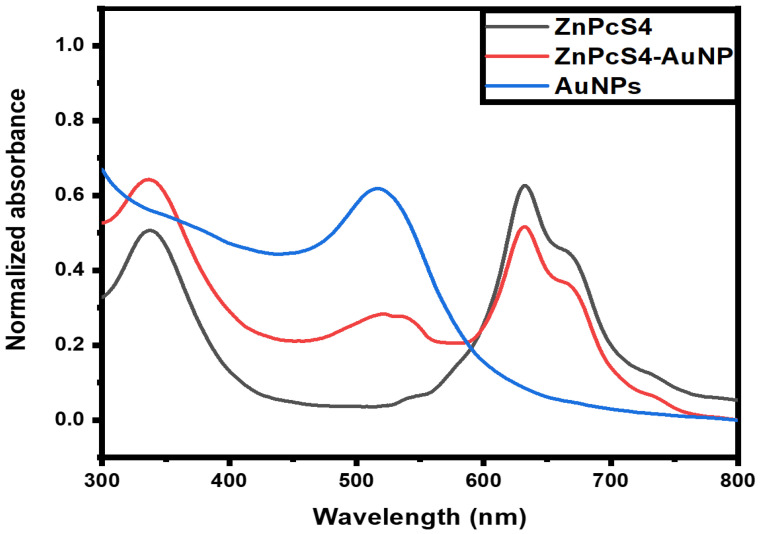
UV-Vis characterisation of ZnPcS_4_-AuNP. The characteristic absorption peaks of AuNPs and ZnPcS_4_ were noted at 526, 350 nm, 634, and 673 nm within the UV-Vis spectrum of the nanoconjugate.

A spectrophotometric analysis was used to confirm the successful binding of the ZnPcS_4_ and AuNPs. The spectroscopic properties of the AuNPs alone, ZnPcS_4_, and the ZnPcS_4_-AuNP nanoconjugate were investigated, as illustrated in Figure 1. With reference to Figure 1, the absorbance spectrum of the ZnPcS_4_ exhibited its three distinctive peaks at 350 nm, 634 nm, and 673 nm, as anticipated [26]. Similarly, the AuNP spectrum demonstrated its characteristic peak at 520 nm. Within the absorbance spectrum of the ZnPcS_4_-AuNP, there were prominent peaks at 350 nm, 634 nm, and 673 nm, which closely corresponded to the presence of ZnPcS_4._ This suggested that the PS was not only bound to the AuNPs, but also retained its spectroscopic properties. Within the same spectrum, there was a slight shift in the resonance peak assigned to the AuNPs, with an absorbance peak at 526 nm, suggesting the successful adsorption of the ZnPcS_4_ to the AuNPs. Furthermore, the resonance peak showed a slight broadening, inferring ultimate bonding between the AuNPs and ZnPcS_4_ due to the increase in molecular size [27]. 

#### 4.1.2. Determination of Loading Capacity Using UV-Vis Spectrophotometry

**Figure 2:**
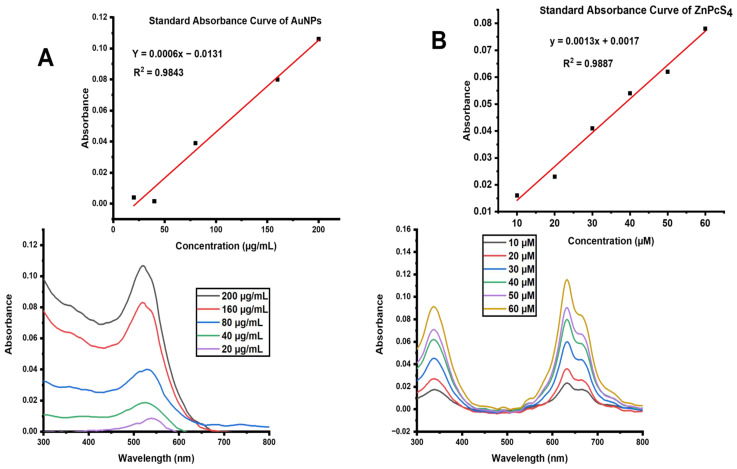
(**A**) UV-Vis spectra of AuNPs with absorbance peaks at 520 nm and a standard curve generated from increasing concentrations of AuNP (20–200 µg/mL). (**B**) UV-Vis spectra of ZnPcS_4_ showing absorption peaks at 350 nm, 634 nm, and 673 nm and the standard curve of ZnPcS_4_ concentrations (10–60 µM).

In order to determine the concentration of the ZnPcS_4_ loaded onto the AuNPs, standard curves illustrating the correlation between the molecules’ measured concentrations and absorbance were plotted for the AuNP and ZnPcS_4_ (Figure 2). The Y = 0.0013x + 0.0017 and Y = 0.0006x − 0.0131 equations, obtained from the respective standard curves of the ZnPcS_4_ and AuNPs, were used to calculate the concentrations of both the ZnPcS_4_ and AuNPs in the nanoconjugate, as shown in Table 1. The initial concentration of the ZnPcS_4_ added to the AuNPs was 125 µM, and only 83 µM was bound. Therefore, using the equation mentioned in Section 2.6, the loading efficiency of ZnPcS_4_ was found to be 66.4%. 

#### 4.1.3. FTIR Analysis 

**Figure 3:**
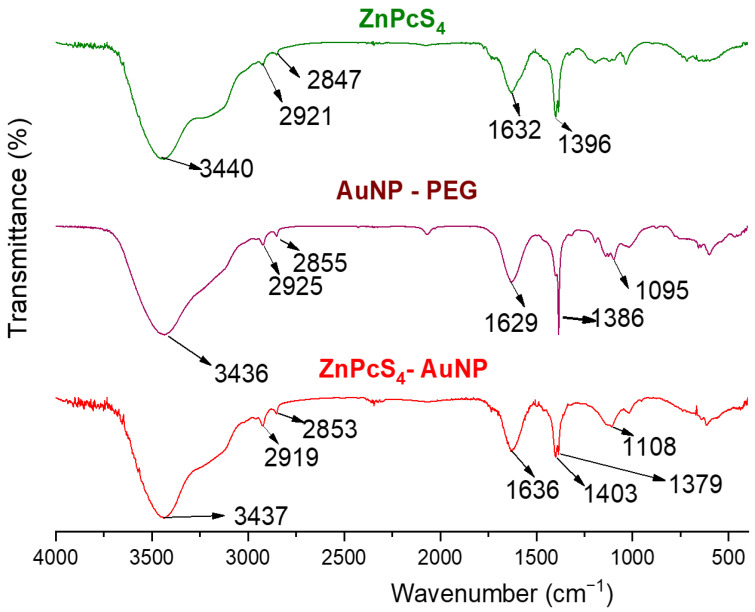
FTIR spectral analysis of AuNPs, ZnPcS_4_, and ZnPcS_4_-AuNP nanoconjugate.

The presence of ZnPcS_4_ and AuNP-PEG in the spectra of the ZnPcS_4_-AuNPs was confirmed using FTIR (Figure 3). Two absorption bands at 1629 and 1386 cm^−1^ in the spectra of the AuNPs pertained to the asymmetric and symmetric stretching of the COO^−^ groups in the sodium citrate coating. The broad O-H bending at 3436 cm^−1^ was due to the NPs being suspended in an aqueous solution and the peaks at 1095 cm^−1^ represented the C-O-C functional group present in SH-PEG-NH_2_. Within the ZnPcS_4_ spectra, absorption peaks at 1396 and 1632 cm^−1^ were related to S=O and C=C functional groups, respectively. Additionally, prominent C-H peaks were observed in all the compounds at 2925–2847 cm^−1^. The peaks at 400–750 cm^−1^ within the ZnPcS_4_-AuNP spectra could be closely related to the bonding of the AuNPs with sulphur (Au-S). Additionally, the absorption peak at 1379 cm^−1^ was related to S=O stretching from the sulfonate groups of the ZnPcS_4_, which confirmed the presence of the PS in the PS-AuNPs. Lastly, slight spectral shifts were noted for the C=O of the conjugate as compared to the PEG-AuNPs, confirming the successful binding of ZnPcS_4_ to the AuNPs.

#### 4.1.4. Particle Size and Morphology

**Figure 4:**
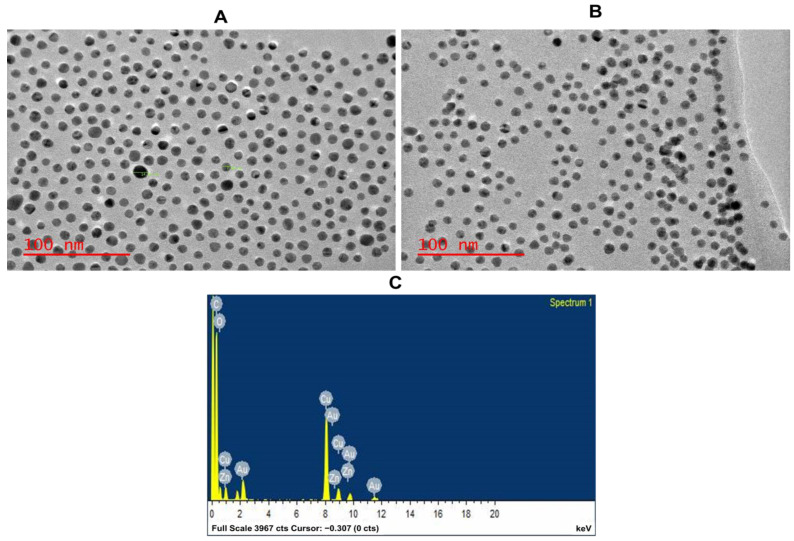
Characterisation of ZnPcS_4_-AuNP nanoconjugate, (**A**) AuNP; (**B**) ZnPcS_4_-AuNP; and (**C**) EDS spectra of ZnPcS_4_-AuNP.

TEM was used to determine the size and morphological appearance of the ZnPcS_4_-AuNPs (Figure 4A,B). The AuNPs appeared to be spherical in shape and had an average size of 14 nm (Figure 3A). No morphological alterations nor agglomeration were detected after loading the ZnPcS_4_ onto the AuNPs (Figure 4B). The AuNPs still retained their initial size, suggesting a remarkable distribution free of aggregation. Furthermore, an EDS analysis confirmed that the ZnPcS_4_-AuNPs were composed of carbon (C), oxygen (O), zinc (Zn), gold (Au), and copper (Cu) elements. However, the Cu atoms were detected on the grids used for the EDS studies, not in the compound.

#### 4.1.5. Zeta Potential and DLS

The nanoconjugate and its components were analysed for their size and zeta potential (Table 2). The hydrodynamic size of the AuNPs was 44.57 nm, while the ZnPcS_4_ and ZnPcS_4_-AuNPs demonstrated average sizes of 53.12 and 61.68 nm, respectively. As anticipated, there was an increase in the size of the nanoconjugate as compared to the AuNPs, suggesting that the ZnPcS_4_ was successfully bound to the AuNPs. Phthalocyanines typically form aggregates by stacking together, which results in an increase in size when conjugated with nanoparticles [28]. As illustrated in Table 1, the polydispersity (PDI) of the AuNPs was found to be 0.251, while that of the ZnPcS_4_ and ZnPcS_4_-AuNP was noted as 0.339 and 0.424, respectively. All the PDI values were indicative of a good size distribution, and a PDI value less than 0.71 would suggest a lower particle size distribution [29]. DLS tends to favour bigger sizes when compared to other techniques; hence, the results reported by TEM were smaller than those obtained from DLS [30].

#### 4.1.6. Cellular Uptake and Release ZnPcS_4_

**Figure 5:**
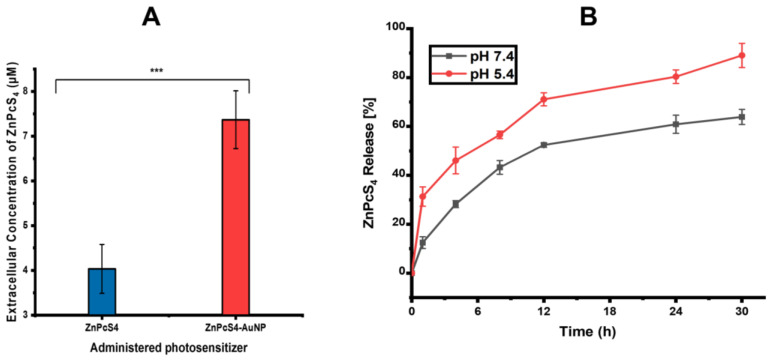
(**A**) Intracellular uptake of ZnPcS_4_ in spheroids treated with the same concentration of ZnPcS_4_ and ZnPcS_4_-AuNP (*p* < 0.001 ***). (**B**) ZnPcS_4_ released from AuNPs into the medium (pH 7.4 or 5.4).

The intracellular uptake of the ZnPcS_4_ was measured after 24 h of incubation of the spheroids with 12.73 µM of free ZnPcS_4_, as well as with the same concentration of ZnPcS_4_ conjugated to the AuNPs. As shown in Figure 5A, approximately 4.03 µM of the administered 12.73 µM of free ZnPcS_4_ was retained in the spheroids after 24 h of incubation, suggesting a poor uptake. The intracellular level of ZnPcS_4_ was found to be significantly higher in the spheroids treated with the same concentration of ZnPcS_4_, However, 7.4 µM was retained when they were conjugated to the AuNPs, versus incubation with the free ZnPcS_4_ (*p* < 0.001 ***). The PS release profiles were evaluated at both pH 7.4 and pH 5.4 to mimic the physiological conditions of the human body and tumour microenvironments, respectively. As observed in Figure 5B, the release of ZnPcS_4_ from the AuNPs at pH 7.4 ranged from 12.5% to 64% after 30 h, whereas a faster ZnPcS_4_ release was noted at pH 5.4, from 31% to 89% after 30 h.

#### 4.1.7. Localisation of ZnPcS_4_-AuNP in MCTS

**Figure 6:**
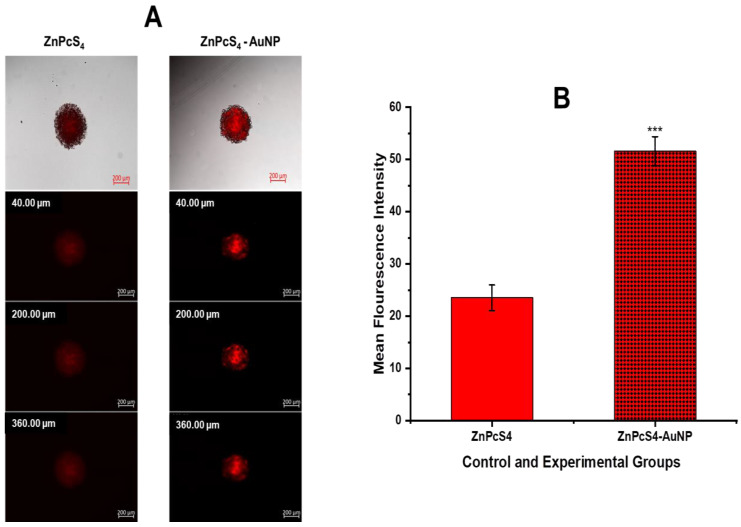
(**A**) Fluorescence images of A375 spheroids after treatment with ZnPcS_4_ and ZnPcS_4_-AuNP; the Z-stack scanning was reported from 40–360 µm depth from the periphery. (**B**) Mean fluorescence intensity of ZnPcS_4_-AuNP compared to that of ZnPcS_4_ (*p* < 0.001 ***).

Fluorescence microscopy was used to determine the distribution and fluorescence intensity of the ZnPcS_4_ in the A375 spheroids at increasing depths from the periphery. In Figure 6A, sections (40–360 µm) from the spheroids treated with the free ZnPcS_4_ demonstrated a varying low level of fluorescent signal throughout the entire cross-section. However, the spheroids treated with the ZnPcS_4_-AuNP exhibited a detectable fluorescence intensity distributed throughout the entire cross-section (though with a varying intensity), and the ZnPcS_4_ accumulated predominantly on the spheroid surface. As observed in Figure 6B, there was a significant increase in the fluorescence intensity of the spheroids treated with ZnPcS_4_-AuNP as compared to the free ZnPcS_4_, which was attributed to the increased cellular uptake of the NPs.

#### 4.1.8. Dose–Response Studies and IC_50_ Concentration Determination

**Figure 7:**
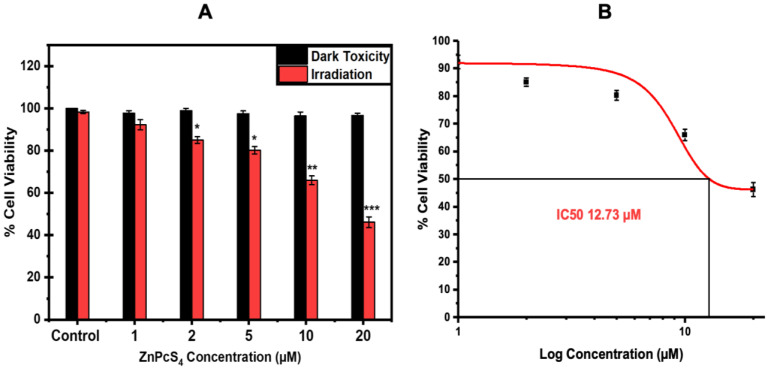
(**A**) A375 spheroids treated with increasing doses of ZnPcS_4_ and photoactivated with 673 nm laser at a fluency of 10 J/cm^2^. The statistical significance is depicted as (*p* < 0.05 *, *p* < 0.01 **, and *p* < 0.001 ***). (**B**) IC50 determination using a sigmoidal graph.

The A375 tumour spheroids were treated with increasing doses of ZnPcS_4_ (1–20 µM) combined with 10 J/cm^2^ (Figure 7A). No photodamage was observed in the spheroids treated with either the ZnPcS_4_ alone or laser irradiation alone. As observed in Figure 7A, the ZnPcS_4_-mediated inhibitory effect on the viability significantly increased proportionally to the rise in concentration, except for the spheroids treated with the photoactivated ZnPcS_4_ at a concentration of 1 µM. According to the PDT results revealed by the MTT assay, the IC_50_ concentration was found to be 12.73 µM using the sigmoidal fitting/dose response curve (origins), which was applied throughout the study (Figure 7B). 

### 4.2. ZnPcS_4_-AuNP-Mediated PDT Treatment

#### 4.2.1. Morphology

##### Inverted Light Microscope

**Figure 8:**
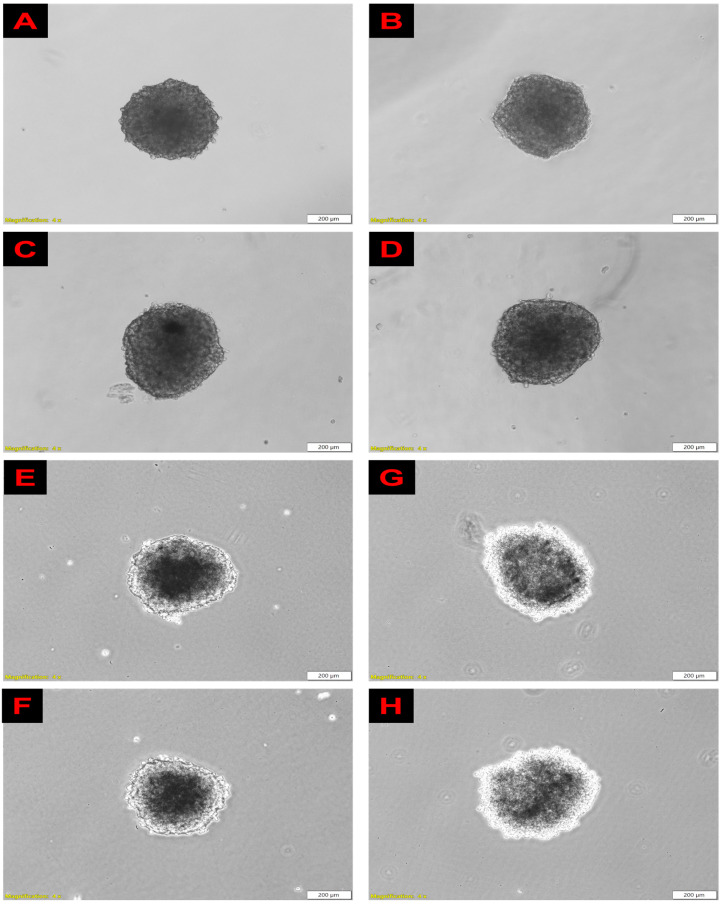
Morphological features of spheroids 24 h post-PDT treatment. (**A**) Cells only; (**B**) Cells + Irradiation; (**C**) AuNPl (**D**) AuNP + Irradiation; (**E**) ZnPcS_4_; (**F**) ZnPcS_4_-AuNP; (**G**) ZnPcS_4_ + Irradiation; and (**H**) ZnPcS_4_-AuNP + Irradiation. Treated spheroids showing a disrupted outer membrane suggesting photodamage.

Morphological images of spheroids were captured 24 h post-PDT treatment. As seen in Figure 8, there were no detectable changes in the structural architecture of the spheroids treated with either the non-irradiated free ZnPcS_4_, conjugated ZnPcS_4_, or laser irradiation alone. Their outer membranes remained intact with a prominent outline. However, the irradiated spheroids that received 12.73 µM of ZnPcS_4_ and ZnPcS_4_-AuNP revealed significant morphological irregularities as compared to the untreated spheroids. The spheroids appeared swollen, lost their prominent outline, and had a disintegrated periphery, which was attributed to cell death in the outer rim of the spheroid.

#### 4.2.2. Live/Dead Assay

**Figure 9:**
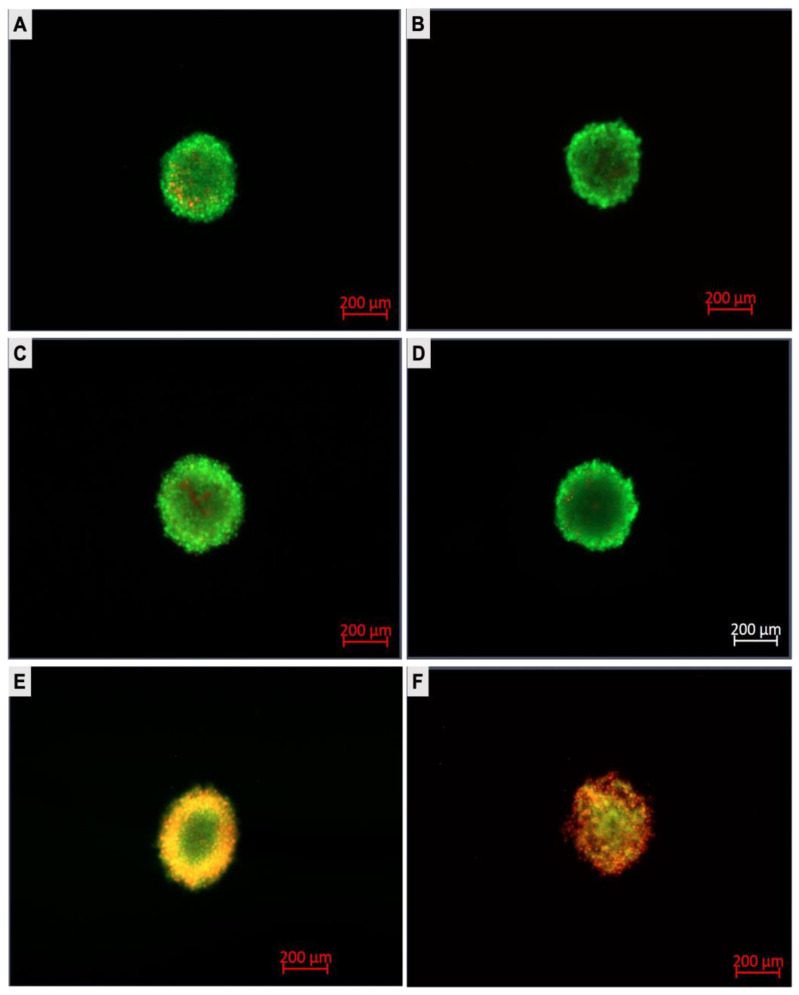
A375 spheroids labelled with AO/EtBr 24 h post-PDT treatment. Live cells stained with AO while photodamaged cells stained the EtBr. (**A**) Cells only; (**B**) Cells + Irradiation; (**C**) ZnPcS_4_; (**D**) ZnPcS_4_-AuNP; (**E**) ZnPcS_4_ + Irradiation; and (**F**) ZnPcS_4_-AuNP Irradiation.

The A375 spheroids were stained with AO/EtBr 24 h post-PDT treatment. Dual staining was visualised under a fluorescent microscope. The AO stain penetrated all cells and caused the nuclei to emit green fluorescence, while EtBr only permeated the cells with disrupted cytoplasmic membranes and stained the nuclei red [31]. With reference to Figure 9, the untreated spheroids (A) did not take up the EtBr staining, suggesting a negative cytotoxicity. Similarly, the spheroids exposed to laser irradiation alone or the bare and conjugated ZnPcS_4_ alone did not stain with EtBr, indicative of an intact cytoplasmic membrane and cell viability. However, the PDT-treated spheroids absorbed the EtBr stain due to a compromised cytoplasmic membrane integrity, with the nanoconjugate-treated spheroids exhibiting the most severe photodamage. 

#### 4.2.3. Cellular Viability

**Figure 10:**
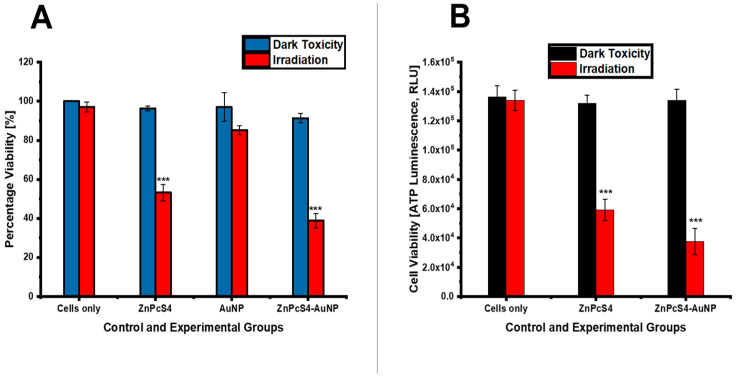
(**A**) Cell viability of spheroids 24 h post-PDT treatment, and (**B**) ATP luminescence 24 h post-PDT treatment (*p* < 0.001 ***).

As seen in Figure 10A, neither the spheroids exposed to laser irradiation alone, AuNPs, nor the bare and conjugated ZnPcS_4_ alone demonstrated any statistically significant decrease in their viability. Similarly, the spheroids treated with the AuNPs and laser irradiation showed no significant changes in their cell viability as compared to the untreated spheroids. However, significant phototoxic effects were noted in the PDT-treated spheroids, with the ZnPcS_4_-AuNPs exerting a significantly greater inhibitory effect (38% ***) when compared to the untreated spheroids. With reference to Figure 10B, a significant level of luminescent signal was detected in the untreated spheroids, which correlated with an increase in ATP production and ultimately an increased metabolic activity. Likewise, the spheroids treated with both the bare and conjugated ZnPcS_4_ alone or laser irradiation alone exhibited considerable levels of luminescent signal. These findings suggested that laser irradiation alone and the inactivated bare or conjugated ZnPcS_4_ exerted no inhibitory effects on the spheroids. In contrast, the spheroids treated with the photoactivated ZnPcS_4_ demonstrated a statistically significant decrease in ATP production as compared to the untreated control spheroids (*p* < 0.001 ***). However, the most potent inhibitory effect was achieved with the ZnPcS_4_-AuNPs, suggesting that the NPs increased the solubility and cellular uptake of the ZnPcS_4_ spheroids.

#### 4.2.4. Cell Death 

**Figure 11:**
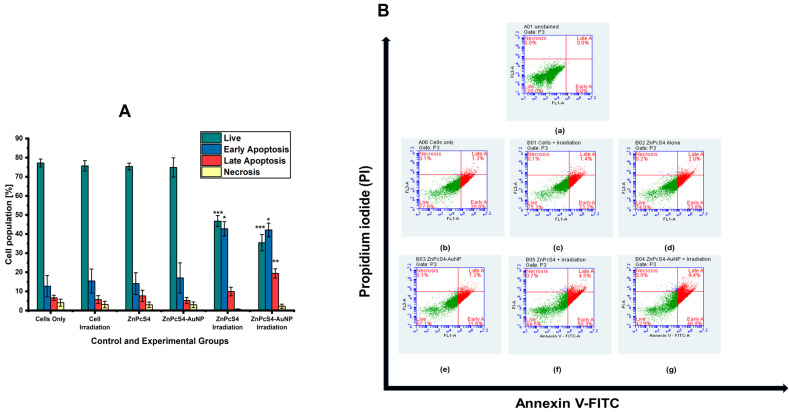
Annexin/PI staining used to determine the mode of cell induction 24 h post-PDT treatment with free ZnPcS_4_ and ZnPcS_4_-AuNP. (**A**) The statistical data of flow results showing mean ± standard error of three independent experiments (*p* < 0.05 *, *p* < 0.01 **, and *p* < 0.001 ***). (**B**) Scattergrams obtained from one of the three independent experiments: (**a**) unstained cells, (**b**) untreated cells only, (**c**) cells + irradiation, (**d**) ZnPcS_4_ alone, (**e**) ZnPcS_4_-AuNP, (**f**) ZnPcS_4_ + irradiation, and (**g**) ZnPcS_4_-AuNP + irradiation.

Figure 11 illustrates the flow cytometry analysis using Annexin V/PI 24 h post-PDT treatment. No substantial changes in cell populations were detected in the spheroids treated with laser irradiation alone or with the non-irradiated bare and conjugated ZnPcS_4_. However, the PDT treatment with the free ZnPcS_4_ considerably reduced the proportion of live cells to 47% ***, whereas 43% * and 10% of the cell populations were in the early and late apoptotic phases, respectively, when compared to the untreated spheroids. The equivalent dose of ZnPcS_4_, however, conjugated to the AuNPs, further reduced the cell viability to 35% *** and significantly increased the early (42% *) and late apoptotic cell populations (19.4% **) when compared to their corresponding population type of the untreated spheroids_._ Figure 11B represents scattergrams obtained from one of three separate experiments. 

#### 4.2.5. Fluorometric Quantification of Caspase-3, 8, and 9 Activities 

**Figure 12:**
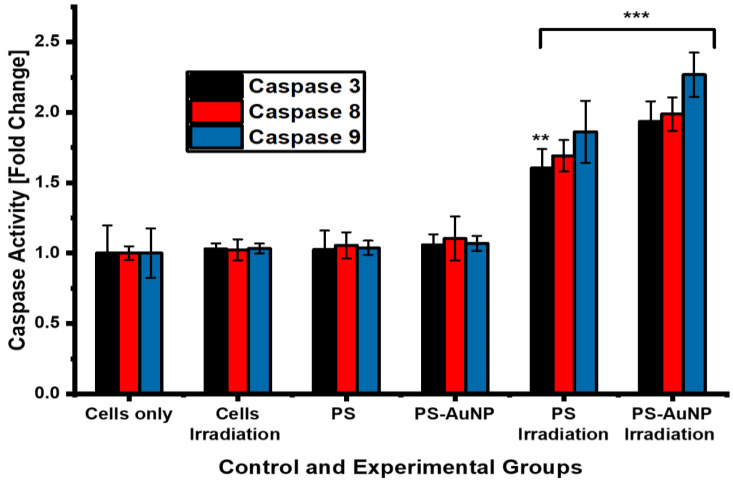
Caspase-3, 8, and 9 levels in ZnPcS_4_-PDT and ZnPcS_4_-AuNP-PDT were determined by fluorometric caspase assay. Results present fold changes relative to untreated controls. Significant difference between control and experimental groups was indicated by *p* < 0.01 **, and *p* < 0.001 ***.

A fluorometric assay was used to determine the activity of the apoptotic proteins, caspase 3, 8, and 9 in the spheroids 24 h after the PDT treatment with the free ZnPcS_4_ and nanoconjugate. As illustrated in Figure 12, there was no statistical difference in the caspase 3, 8, and 9 levels of the spheroids subjected to laser irradiation alone, the bare ZnPcS_4_, or the nanoconjugate alone when compared to the untreated control cells. However, significant statistical differences in the caspase 3, 8, and 9 expressions were found in the irradiated spheroids that received ZnPcS_4_, with 1.6 **, 1.69 ***, and 1.89 *** fold changes, respectively, relative to the untreated control cells. Furthermore, a slight improvement in the caspase 3, 8, and 9 levels was observed after the PDT treatment with the ZnPcS_4_-AuNPs, with 1.94-, 1.99-, and 2.27-fold increases with respect to the untreated control cells (*p* < 0.001 ***).

## 5. Discussion

In recent years, PDT has gained popularity in the eradication of various cancers, such as melanoma, colon cancer, and lung cancer, due to its increased potency and negligible side effects on normal tissues [28]. However, a series of limitations relating to a poor solubility, off-target toxicity, and eminent aggregation under physiological conditions have drastically hampered the effectiveness of classical PDT [8]. To tackle this issue, various nanoparticles, such as gold nanoparticles, have been widely used in PDT to enhance the PS delivery in cancer cells in order to promote more robust inhibitory effects and improved clinical outcomes. However, PDT studies are typically conducted on 2D cell culture models, which fail to recapture the tumour microenvironment [14]. In the present work, we synthesised a nanoparticle-mediated PS delivery system comprising ZnPcS_4_ conjugated to polyethylene glycol (PEG)-functionalised gold nanoparticles to form ZnPcS_4_-AuNPs and investigated their cytotoxic effect on 3D melanoma spheroids to bridge the gap between in vitro and in vivo studies.

The nanoconjugate was successfully synthesised and subjected to elementary characterisation techniques. A UV/visible analysis confirmed that all the distinctive absorption peaks of the ZnPcS_4_ and AuNPs were detected within the spectra of the ZnPcS_4_. A similar observation was made when looking at the FTIR spectra of the nanoconjugate. Studies by Montaseri et al. reported that ZnPcS_4_ PS was adsorbed onto the surface of Ag-S-PEG-NH_2_ via potential interactions of hydrogen bonding, van de Waals forces, and electrostatic interactions, suggesting a possible interaction in this current study [5]. Furthermore, the nanoconjugate exhibited a PDI value of 0.424 nm at 61.68 nm, indicating its remarkable distribution with no agglomeration [32]. The nanoconjugate attained a ZP value of −18.8 mV, suggesting moderate stability that allowed for passive cellular uptake and selective retention in cancer cells [33,34]. Negatively charged nanoparticles demonstrate an enhanced diffusion and can serve as excellent drug delivery systems for delivering anticancer drugs into deeper tissues [35,36]. Furthermore, they are able to evade the adsorption of serum proteins, which prolongs their in vivo half-lives [35]. In relation to Figure 5A and Figure 6, the nanoconjugate showed an increased cellular uptake and fluorescence intensity, respectively, when compared to the bare ZnPcS_4_. This was due to the fact that second-generation PSs are inherently hydrophobic, which drastically affects their bioavailability in targeted regions. Therefore, their modification with nanoparticles increases their solubility and cellular uptake, since nanoparticles are hydrophilic in nature and have a high loading capacity [4]. Furthermore, the improved cellular uptake and distribution of the ZnPcS_4_ in the spheroids treated with the nanoconjugate could also be attributed to the small size of the AuNPs, which allows them to penetrate into deeper regions of tumour cells [37]. Through further analysis, it was observed that the release of ZnPcS_4_ from the AuNPs was faster at pH 5.4 compared to pH 7.4, suggesting that AuNPs could release PSs in a well-coordinated manner upon exposure to an acidic endo/lysosomal pH and intracellular reductive conditions in tumour cells. Similar observations were reported by Shahidi et al., who highlighted that AuNPs could effectively penetrate cancer cells and control the release of therapeutic drugs, thereby preventing unwanted drug leakage before reaching targeted tumour sites [38].

ZnPcS_4_ noted an IC50 concentration of 12.73 µM in the A375 spheroids, which was five times higher than that by reported in previous studies by Naidoo et al. [39]. This reduced responsiveness to PDT was attributed to the fact that tumour spheroids closely embody the hypoxic conditions and cellular interactions of solid tumours [40]. The treatment with laser irradiation alone had negligible effects on the morphological features, cellular viability, and proliferation of the A375 monolayers. Studies by Manoto et al. [41] also demonstrated that irradiation alone at 10 J/cm^2^ did not alter the morphological features, cell viability, and proliferation of lung cancer spheroids. Likewise, the A375 spheroids treated with either the free ZnPcS_4_ or nanoconjugate alone showed no impacts on their cellular morphology, viability, and proliferation. These results suggest that ZnPcS_4_ has no dark toxicity, which makes it an ideal PS for PDT treatment. Significant morphological alterations were observed following the irradiation of the spheroids incubated with the free ZnPcS_4_ and nanoconjugate. The spheroids lost their integrity and clear outline, which signified photodamage in the outer layers of the spheroid. These findings concurred with morphological changes reported by Sokolova et al. and Cogno et al. [42,43] in PDT-treated spheroids. Furthermore, the live/dead assay in Figure 5 revealed an increased fluorescence intensity of the EtBr stain in the irradiated spheroids, with the ZnPcS4-AuNP-mediated PDT exhibiting a severe loss of membrane integrity, suggesting an enhanced cytotoxicity. When compared to the PDT treatment with the free PS, the spheroids treated with the nanoconjugate suffered the most severe phototoxicity according to the MTT and ATP assays. These findings coincided with studies conducted by Barbugli et al., which highlighted that, when PSs are modified with nanoparticles, they are more effectively taken up by tumour spheroids and untimely induce appreciable anticancer effects that free the PSs [44].

For a better understanding of the phototoxic mechanisms triggered by the PDT treatment of the A375 spheroids, a flow cytometric analysis was conducted. The control and experimental groups were subjected to Annexin V-FITC and PI for apoptosis and necrosis detection, respectively. The flow cytometric results revealed that the spheroids subjected to the laser irradiation alone, ZnPcS_4_, and nanoconjugate alone still retained a high proportion of viable cell populations as compared to the untreated spheroids. However, when comparing the same group to the spheroids subjected to the laser irradiation and ZnPcS_4_, a noticeable reduction in the proportion of viable cells (47% ***) was noted. Furthermore, there was also a substantial increase in the proportion of early apoptotic cells (43% *), while both the proportions of late apoptotic and necrotic cells remained insignificant. These results are in agreement with the studies performed by Manoto et al., who also noted that photoactivated ZnPcS_mix_ administered to tumour spheroids significantly reduced the viable cell population, as well as increased the early apoptosis cell population [45].

The most substantial inhibitory effects were inflicted by the ZnPcS_4_-AuNPs, which resulted in a significant reduction in the proportion of viable cells (35% ***), with markedly increased proportions of the early and late apoptotic cell populations of 42% * and 19.4% **, respectively. These findings suggest that the ZnPcS_4_-AuNP-mediated PDT annihilated the melanoma cells via the induction of apoptosis, which concurs with studies reported by Naidoo et al., 2019 [39]. This particular cell death mechanism can be initiated by two pathways, namely the intrinsic and extrinsic pathways [46]. PDT typically triggers the release of mitochondrial cytochrome c into the cytosol, which, in turn, forms an apoptosme complex that promotes the activation of caspase-9 (the initiator) and caspase-3 (the effector). However, studies have reported that caspase 9 can be activated in the presence of caspase 8 to prompt caspase-3 for apoptosis execution [47]. In relation to Figure 12, the PDT treatment with the ZnPcS_4_-AuNPs showed more pronounced levels of initiator caspase 8 (extrinsic), 9 (intrinsic), and 3 (executioner) activities than the free ZnPcS_4_, with the caspase-9 levels being slightly elevated in all the PDT-treated spheroids. These findings aligned with reports that have stated that apoptosis is a diverse pathway that may incorporate both the mitochondrial and death receptor pathways [47]. A similar observation was reported by Doustvandi et al. (2019), following the ZnPcS_4_-mediated PDT treatment of skin cancer cells [48]. The overall enhanced cytotoxicity of the nanoconjugate, when compared to the free PS, was indicative that the AuNPs increased the passive cellular uptake via the EPR effect, which increased the bioavailability in the cancer cells, as described by Hong et al. [8]. In addition, studies by Dube et al. [49] pointed out that phthalocyanines modified with AuNPs augment triplet state and singlet oxygen quantum yields and ultimately improve PDT activity. Despite showing more robust inhibitory effects on the unresponsive A375 spheroids than the free PS, ZnPcS_4_-AuNPs require additional alterations to improve their PDT-mediated antitumor abilities. In view of this, recent studies call for the further functionalisation of PS nanocarriers with targeting moieties such as antibodies, peptides, and aptamers to increase the affinity and cellular uptake of PSs in cancer cells, as well as mitigate their off-target toxicity [50].

## Figures and Tables

**Table 1:** Estimated concentrations of ZnPcS_4_ and AuNPs on the nanoconjugate.

Sample	Absorbance	Equation	Estimated Concentration in the Nanoconjugate
ZnPcS_4_	0.11	Y = 0.0013x + 0.0017	83 µM
AuNPs	0.09	Y = 0.0006x − 0.0131	172 µg/mL

**Table 2:** DLS, PDI, and Zeta potential measurements.

Sample	DLS (d.nm)	PDI	Zeta Potential (MV)
AuNP	44.57	0.251	−5.70
ZnPcS_4_	53.12	0.339	−9.46
ZnPcS_4_-AuNP	61.68	0.424	−18.8

## Data Availability

Not applicable.
